# 5,11-Diisopropyl-2,8-dimethyl-1*H*,7*H*-diimidazo[*c*,*h*][1,6]diazecine dihydrate

**DOI:** 10.1107/S1600536810025808

**Published:** 2010-07-10

**Authors:** Guillermo Mendoza-Díaz, María de Lourdes Betancourt-Mendiola, Sylvain Bernès

**Affiliations:** a Universidad de Guanajuato, División de Ciencias e Ingeniería, Departamento de Ingeniería Física, Lomas del Bosque No. 103, Col. Lomas del Campestre, 37150 Leon, Gto., Mexico; b Universidad de Guanajuato, División de Ciencias Naturales y Exactas, Departamento de Química, Noria Alta s/n, 36050 Guanajuato, Gto., Mexico; c DEP Facultad de Ciencias Químicas, UANL, Guerrero y Progreso S/N, Col. Treviño, 64570 Monterrey, N.L., Mexico

## Abstract

Crystals of the title compound, C_18_H_30_N_6_·2H_2_O, are composed of units of diimidazo[*c*,*h*][1,6]diazecine and two water mol­ecules. The asymmetric unit contains one half-molecule of diazecine and one uncoordinated water molecule in a general position. The complete ten-membered heterocycle is generated by an inversion center.The organic residue and water mol­ecules form a two-dimensional hydrogen-bonded network. The 1,6-diazecine ring shows a chair conformation, with angles and distances in normal ranges.

## Related literature

For background to imidazoles, see: Bouwman *et al.* (1990 ▶). For the Mannich reaction, see: Stocker *et al.* (1970 ▶). The reaction of formaldehyde, or other aldehydes, with different substrates and conditions has been widely used in organic synthesis, see: Teo *et al.* (1993 ▶); Berndt (1970 ▶); Geue *et al.* (1994 ▶); Karunakaran & Kandaswamy (1994 ▶); Baumann *et al.* (1984 ▶); Stocker *et al.* (1970 ▶); Mendoza-Díaz *et al.* (1996 ▶). For a similar structure, see: Mendoza-Díaz *et al.* (2002 ▶). For the structures of copper complexes with different diazecine derivatives, see: Gasque, Mijangos & Ortiz-Frade (2005 ▶); Gasque, Olguín & Bernès (2005 ▶); Luna-Ramírez *et al.* (2008 ▶). 
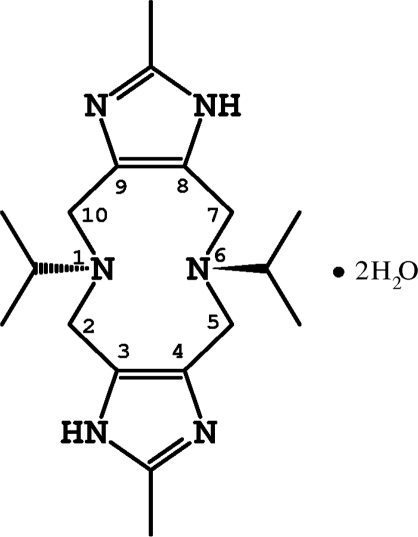


## Experimental

### 

#### Crystal data

C_18_H_30_N_6_·2H_2_O
*M* *_r_* = 366.51
Orthorhombic, 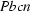

*a* = 12.571 (3) Å
*b* = 14.665 (3) Å
*c* = 10.877 (4) Å
*V* = 2005.3 (9) Å^3^
*Z* = 4
Mo *K*α radiation
μ = 0.08 mm^−1^
*T* = 300 K
0.6 × 0.2 × 0.2 mm

#### Data collection

Siemens P4 diffractometer
2495 measured reflections
1768 independent reflections
1126 reflections with *I* > 2σ(*I*)
*R*_int_ = 0.022
3 standard reflections every 97 reflections  intensity decay: 1%

#### Refinement

*R*[*F* ^2^ > 2σ(*F* ^2^)] = 0.056
*wR*(*F* ^2^) = 0.157
*S* = 1.72
1768 reflections
131 parameters
H atoms treated by a mixture of independent and constrained refinement
Δρ_max_ = 0.19 e Å^−3^
Δρ_min_ = −0.24 e Å^−3^

### 

Data collection: *XSCANS* (Siemens, 1996 ▶); cell refinement: *XSCANS*; data reduction: *XSCANS*; program(s) used to solve structure: *SHELXS97* (Sheldrick, 2008 ▶); program(s) used to refine structure: *SHELXL97* (Sheldrick, 2008 ▶); molecular graphics: *Mercury* (Macrae *et al.*, 2006 ▶), *PLUTON* (Spek, 1992 ▶, 1993 ▶) and *PLATON* (Spek, 2009 ▶); ; software used to prepare material for publication: *SHELXL97*.

## Supplementary Material

Crystal structure: contains datablocks I, global. DOI: 10.1107/S1600536810025808/pb2033sup1.cif

Structure factors: contains datablocks I. DOI: 10.1107/S1600536810025808/pb2033Isup2.hkl

Additional supplementary materials:  crystallographic information; 3D view; checkCIF report

## Figures and Tables

**Table 1:** Hydrogen-bond geometry (Å, °)

*D*—H⋯*A*	*D*—H	H⋯*A*	*D*⋯*A*	*D*—H⋯*A*
N1—H1⋯O1^ii^	0.92 (2)	1.94 (2)	2.815 (3)	159 (2)
O1—H11⋯N3^iii^	0.99 (3)	1.91 (3)	2.890 (3)	169 (3)
O1—H12⋯N7^iv^	0.94 (3)	2.07 (3)	2.943 (2)	155 (3)

Symmetry codes: (ii) 
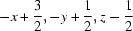
; (iii) 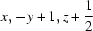; (iv) 
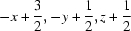
.

**Fig. 1.**
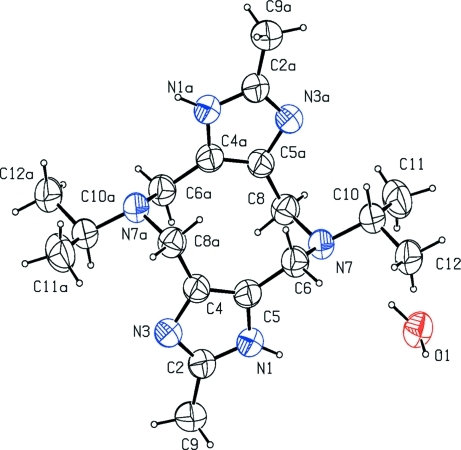
ORTEP plot (Spek, 2009) of isopromeim, showing the atomic numbering scheme. Observe that the diazecine ring is in a chair conformation.

**Fig. 2.**
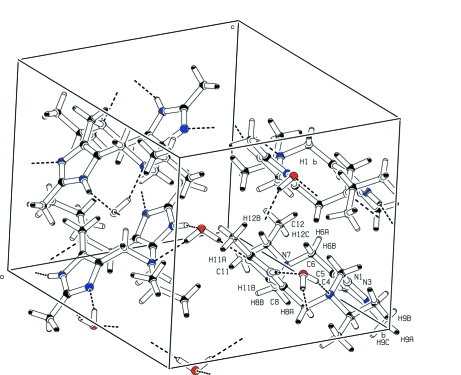
PLUTON98 (Spek, 1992, 1993) plot showing a partial view of the hydrogen bond network in the isopromeim crystal.

## supplementary crystallographic information

### Comment

Imidazole chemistry is of great interest because their wide occurrences in biological systems like a component in the aminoacid histidine or other metabolites as histamine. Also, synthetic imidazole derivatives have found useful in medicine as antihistaminic, antielmitic, antifungiic or amebicide drugs, some of these compounds are 4-disubstituted aminomethyl imidazoles and has been prepared for different multistep process.

The development of new imidazole containing compounds, that could help in the understanding of their properties and the role that imidazole plays in different enzymes, it has been of great interests since many years (Bouwman *et al.*,  1990). Therefore, the continuous develop for easy synthetic processes to obtain new imidazole derivatives is a matter of permanent research.

Mannich reaction is one of those powerful and easy reactions. This reaction is a one-step method to attach aminomethyl groups to the imidazole ring (Stocker *et al.*, 1970). The reaction of formaldehyde, or other aldehydes, with different substrates and conditions has been widely used in organic synthesis. Some of these reactions involve coordinated amino acids (Teo *et al.*, 1993; Berndt, 1970), coordinate amines (Geue *et al.*, 1994), aromatic rings and secondary amines (Karunakaran & Kandaswamy, 1994), 2,4(5)disubtituted imidazoles and secondary amines (Baumann *et al.*, 1984; Stocker *et al.*, 1970). Previously, we have used this reaction under basic conditions to condense propylamine, formaldehyde and 2-methylimidazole (Mendoza-Díaz, Driessen & Reedijk, 1996). The product obtained by the double addition of the formaldehyde on the imidazole ring at the 4 and 5 positions was the heterocyclic 10 member ring: 1,6-diazecine with the imidazole rings fused through the bond between the carbons 4 and 5. In the crystal structure of that compound, an interesting hydrogen bond network was observed, where a cluster of six water molecules in a flat-hexagonal arrangement is in between the diazecine molecules, making a three dimensional network. Also, crystal structure of a similar compound, where instead of propyl substituent an ethanoic acid residue is present has been reported, (Mendoza-Díaz, Driessen, Reedijk, Gorter, Gasque & Thomson, 2002). In this case the water molecules conform an ice-type ring that binds the organic residue in a two dimensional network. Several crystal structures of copper complexes with different diazecine derivatives has been reported (Gasque, Mijangos & Ortiz-Frade, 2005), (Gasque, Olguín & Bernès, 2005), (Luna-Ramírez, Bernès & Gasque, 2008). In all cases the chair conformation of the diazecine ring has been observed.

In this paper, we report the crystal and molecular structure of the title compound, (hereafter referred as isopromeim).

The *ORTEP* plot showing the molecular structure of isopromeim is shown in figure 1 and bond lengths and angles are in the table of selected geometrical data. The 2-methyl group and the methylene carbons attached directly to the imidazole ring are essentially in the same least squere plane of the imidazole. A slight twist of the C(6) and C(8a) is observed due to their participation in the 1,6-diazecine ring. These deviations from the least squares plane of the imidazole are 0.068 (2) and 0.046 (2) Å, respectively; whereas for the 2-methyl group is -0.009 (3) Å out of plane. These values indicate that there is a slight difference with the n-propyl derivative previously reported. The angle formed between the bond C(5)—C(6) and the least squares plane of the imidazole is 2.71 (15)° and for the bond C(4)—C(8a) and the same plane is only of 1.77 (15)°, whereas for the bond between the methyl group and the imidazole ring (the bond C(2)—C(6)) is 0.49 (18)°.

The 10-member ring of the 1,6-diazecine has a chair -type conformation, linking the two imidazole rings. The angles at the nitrogen bridge in the 1,6-diazecine sum 334.6° indicating a pyramidal conformation.

Isopromeim crystals consist of units of the title compound and two water molecules. These water molecules form a two dimensional network along the crystallographic planes (010) and (100). Particularly, these water molecules link the molecules of isopromeim in a two dimensional structure, as it is illustrated in figure 2. In this hydrogen-bond-bridge are involved the corresponding waters molecules and the nitrogen atoms of the organic residue. Water molecule is acting as a proton-donor and also as a proton-acceptor.

The differences between crystal packing of the propyl and isopropyl derivative indicates that even the diazecine ring is essentially the same in these compounds. Residues attached will drive and control the way that water crystallize. This finding could help in the understanding of how in some imidazole-enzymes may activate water for hydrolysis processes.

### Experimental

4.10 g (0.05 mol) of 2-methyl-imidazole and 3.01 g (0.05 mol) of iso-propylamine were added into 20 ml of distilled water. Into this mixture, a solution of 3.13 g of *para*-formaldehyde, (0.1 mol)previously dissolved in 20 ml of water was slowly added with stirring. The pH was adjusted between 11–12 and then it was allowed 8 days under stirring. A first microcrystalline white precipitated was filtered off and washed with cold water. Yield 3.56 g (14%). The remain solution was allowed to slow evaporation, after two weeks a second crop of a crystalline product was isolated, washed with cold water and dried in air, yielding another 7.35 g (total yield 51%). The two crops were dissolved together into 25 ml of hot methanol and then a small amount of water (about 1 ml) was added. The final solution was cooled in the refrigerator. After three weeks colorless crystals with needle shape and well formed were filtered off. Single crystals were selected for X-ray data collection.

### Refinement

The deviation of the goodness-of-fit from the expected value s=1 arises from systematic errors due to a poor alignment of the crystal on the goniometer, as also reflected in rather high standard uncertainties for cell parameters. However, althoug final s.u.'s for positional and displacement parameters are probably underestimated, reported bond lenghts and angles seem to be reliable.

### Crystal data

**Table:**

C_18_H_30_N_6_·2H_2_O	*F*(000) = 800
*M**_r_* = 366.51	*D*_x_ = 1.214 Mg m^−^^3^
Orthorhombic, *P**b**c**n*	Mo *K*α radiation, λ = 0.71073 Å
Hall symbol: -P 2n 2ab	Cell parameters from 100 reflections
*a* = 12.571 (3) Å	θ = 5.0–12.2°
*b* = 14.665 (3) Å	µ = 0.08 mm^−^^1^
*c* = 10.877 (4) Å	*T* = 300 K
*V* = 2005.3 (9) Å^3^	Needle, colourless
*Z* = 4	0.6 × 0.2 × 0.2 mm

### Data collection

**Table:**

Siemens P4 diffractometer	*R*_int_ = 0.022
Radiation source: fine-focus sealed tube	θ_max_ = 25.0°, θ_min_ = 2.1°
graphite	*h* = −2→14
ω scans	*k* = −17→1
2495 measured reflections	*l* = −1→12
1768 independent reflections	3 standard reflections every 97 reflections
1126 reflections with *I* > 2σ(*I*)	intensity decay: 1%

### Refinement

**Table:**

Refinement on *F*^2^	Secondary atom site location: difference Fourier map
Least-squares matrix: full	Hydrogen site location: inferred from neighbouring sites
*R*[*F*^2^ > 2σ(*F*^2^)] = 0.056	H atoms treated by a mixture of independent and constrained refinement
*wR*(*F*^2^) = 0.157	*w* = 1/[σ^2^(*F*_o_^2^) + (0.05*P*)^2^] where *P* = (*F*_o_^2^ + 2*F*_c_^2^)/3
*S* = 1.72	(Δ/σ)_max_ < 0.001
1768 reflections	Δρ_max_ = 0.19 e Å^−^^3^
131 parameters	Δρ_min_ = −0.24 e Å^−^^3^
0 restraints	Extinction correction: *SHELXL97* (Sheldrick, 2008), Fc^*^=kFc[1+0.001xFc^2^λ^3^/sin(2θ)]^-1/4^
Primary atom site location: structure-invariant direct methods	Extinction coefficient: 0.027 (3)

### Fractional atomic coordinates and isotropic or equivalent isotropic displacement parameters (Å^2^)

**Table:**

	*x*	*y*	*z*	*U*_iso_*/*U*_eq_	
N1	0.78914 (15)	0.45225 (13)	−0.09908 (19)	0.0590 (6)	
H1	0.774 (2)	0.3945 (16)	−0.128 (2)	0.071*	
C2	0.7545 (2)	0.53228 (16)	−0.1446 (2)	0.0593 (7)	
N3	0.80031 (14)	0.60155 (12)	−0.08879 (17)	0.0572 (6)	
C4	0.86930 (18)	0.56315 (14)	−0.0031 (2)	0.0522 (6)	
C5	0.86320 (18)	0.47026 (14)	−0.0089 (2)	0.0539 (6)	
C6	0.91226 (18)	0.39548 (14)	0.0627 (2)	0.0569 (6)	
H6A	0.9597	0.4214	0.1237	0.068*	
H6B	0.8568	0.3625	0.1060	0.068*	
N7	0.97232 (14)	0.33121 (11)	−0.01421 (16)	0.0529 (5)	
C8	1.06008 (17)	0.38073 (15)	−0.0766 (2)	0.0570 (6)	
H8A	1.0291	0.4204	−0.1384	0.068*	
H8B	1.1038	0.3364	−0.1193	0.068*	
C9	0.6734 (2)	0.53789 (17)	−0.2431 (3)	0.0764 (8)	
H9A	0.6610	0.6007	−0.2637	0.115*	
H9B	0.6083	0.5107	−0.2151	0.115*	
H9C	0.6984	0.5059	−0.3145	0.115*	
C10	1.00890 (19)	0.25037 (14)	0.0567 (2)	0.0611 (7)	
H10A	1.0537	0.2714	0.1248	0.073*	
C11	1.0735 (2)	0.18781 (17)	−0.0234 (3)	0.0848 (9)	
H11A	1.1404	0.2161	−0.0423	0.127*	
H11B	1.0355	0.1760	−0.0983	0.127*	
H11C	1.0859	0.1314	0.0191	0.127*	
C12	0.9152 (2)	0.19821 (17)	0.1096 (3)	0.0739 (8)	
H12A	0.8770	0.2366	0.1658	0.111*	
H12B	0.9405	0.1452	0.1522	0.111*	
H12C	0.8687	0.1798	0.0441	0.111*	
O1	0.70233 (15)	0.23151 (12)	0.32676 (16)	0.0700 (6)	
H11	0.742 (2)	0.284 (2)	0.362 (3)	0.105*	
H12	0.639 (2)	0.2290 (19)	0.372 (3)	0.105*	

### Atomic displacement parameters (Å^2^)

**Table:**

	*U*^11^	*U*^22^	*U*^33^	*U*^12^	*U*^13^	*U*^23^
N1	0.0540 (12)	0.0480 (12)	0.0750 (14)	−0.0032 (10)	−0.0048 (11)	0.0004 (10)
C2	0.0537 (14)	0.0502 (14)	0.0739 (15)	0.0007 (12)	−0.0024 (12)	−0.0009 (12)
N3	0.0513 (11)	0.0508 (12)	0.0694 (13)	0.0023 (9)	−0.0020 (10)	0.0001 (10)
C4	0.0485 (12)	0.0487 (12)	0.0594 (13)	0.0030 (11)	0.0034 (12)	0.0008 (11)
C5	0.0471 (12)	0.0533 (14)	0.0614 (13)	−0.0013 (11)	0.0040 (12)	0.0021 (12)
C6	0.0577 (13)	0.0527 (13)	0.0601 (13)	0.0022 (11)	0.0065 (12)	0.0038 (11)
N7	0.0569 (10)	0.0459 (10)	0.0560 (10)	0.0003 (9)	−0.0004 (9)	0.0033 (9)
C8	0.0585 (14)	0.0544 (13)	0.0580 (13)	−0.0003 (11)	0.0030 (12)	−0.0022 (11)
C9	0.0684 (16)	0.0679 (16)	0.0928 (18)	−0.0031 (13)	−0.0230 (15)	0.0016 (15)
C10	0.0640 (14)	0.0486 (13)	0.0705 (16)	−0.0009 (12)	−0.0115 (13)	0.0066 (11)
C11	0.0915 (19)	0.0565 (15)	0.107 (2)	0.0182 (15)	0.0023 (18)	0.0028 (16)
C12	0.0824 (17)	0.0592 (15)	0.0801 (17)	−0.0076 (14)	−0.0075 (15)	0.0177 (14)
O1	0.0766 (13)	0.0569 (11)	0.0765 (12)	−0.0053 (9)	0.0098 (10)	−0.0044 (9)

### Geometric parameters (Å, °)

**Table:**

N1—C2	1.346 (3)	C8—H8B	0.9700
N1—C5	1.378 (3)	C9—H9A	0.9600
N1—H1	0.92 (2)	C9—H9B	0.9600
C2—N3	1.316 (3)	C9—H9C	0.9600
C2—C9	1.481 (4)	C10—C11	1.504 (3)
N3—C4	1.392 (3)	C10—C12	1.518 (3)
C4—C5	1.366 (3)	C10—H10A	0.9800
C4—C8^i^	1.489 (3)	C11—H11A	0.9600
C5—C6	1.480 (3)	C11—H11B	0.9600
C6—N7	1.469 (3)	C11—H11C	0.9600
C6—H6A	0.9700	C12—H12A	0.9600
C6—H6B	0.9700	C12—H12B	0.9600
N7—C8	1.485 (3)	C12—H12C	0.9600
N7—C10	1.487 (3)	O1—H11	0.99 (3)
C8—C4^i^	1.489 (3)	O1—H12	0.94 (3)
C8—H8A	0.9700		
			
C2—N1—C5	108.25 (19)	H8A—C8—H8B	107.3
C2—N1—H1	127.6 (16)	C2—C9—H9A	109.5
C5—N1—H1	123.7 (16)	C2—C9—H9B	109.5
N3—C2—N1	111.2 (2)	H9A—C9—H9B	109.5
N3—C2—C9	126.3 (2)	C2—C9—H9C	109.5
N1—C2—C9	122.5 (2)	H9A—C9—H9C	109.5
C2—N3—C4	105.62 (19)	H9B—C9—H9C	109.5
C5—C4—N3	109.7 (2)	N7—C10—C11	110.64 (19)
C5—C4—C8^i^	127.7 (2)	N7—C10—C12	110.98 (19)
N3—C4—C8^i^	122.53 (19)	C11—C10—C12	109.4 (2)
C4—C5—N1	105.2 (2)	N7—C10—H10A	108.6
C4—C5—C6	133.7 (2)	C11—C10—H10A	108.6
N1—C5—C6	120.95 (19)	C12—C10—H10A	108.6
N7—C6—C5	112.94 (18)	C10—C11—H11A	109.5
N7—C6—H6A	109.0	C10—C11—H11B	109.5
C5—C6—H6A	109.0	H11A—C11—H11B	109.5
N7—C6—H6B	109.0	C10—C11—H11C	109.5
C5—C6—H6B	109.0	H11A—C11—H11C	109.5
H6A—C6—H6B	107.8	H11B—C11—H11C	109.5
C6—N7—C8	109.17 (15)	C10—C12—H12A	109.5
C6—N7—C10	112.00 (16)	C10—C12—H12B	109.5
C8—N7—C10	113.41 (18)	H12A—C12—H12B	109.5
N7—C8—C4^i^	116.58 (19)	C10—C12—H12C	109.5
N7—C8—H8A	108.1	H12A—C12—H12C	109.5
C4^i^—C8—H8A	108.1	H12B—C12—H12C	109.5
N7—C8—H8B	108.1	H11—O1—H12	105 (2)
C4^i^—C8—H8B	108.1		
			
C5—N1—C2—N3	−0.7 (3)	C2—N1—C5—C6	177.1 (2)
C5—N1—C2—C9	−179.6 (2)	C4—C5—C6—N7	−123.6 (3)
N1—C2—N3—C4	0.6 (3)	N1—C5—C6—N7	61.1 (3)
C9—C2—N3—C4	179.4 (2)	C5—C6—N7—C8	61.0 (2)
C2—N3—C4—C5	−0.2 (3)	C5—C6—N7—C10	−172.52 (18)
C2—N3—C4—C8^i^	177.6 (2)	C6—N7—C8—C4^i^	51.4 (2)
N3—C4—C5—N1	−0.2 (3)	C10—N7—C8—C4^i^	−74.2 (2)
C8^i^—C4—C5—N1	−177.9 (2)	C6—N7—C10—C11	−177.84 (18)
N3—C4—C5—C6	−176.1 (2)	C8—N7—C10—C11	−53.7 (2)
C8^i^—C4—C5—C6	6.2 (4)	C6—N7—C10—C12	60.6 (2)
C2—N1—C5—C4	0.6 (3)	C8—N7—C10—C12	−175.3 (2)

Symmetry codes: (i) −*x*+2, −*y*+1, −*z*.

### Hydrogen-bond geometry (Å, °)

**Table:**

*D*—H···*A*	*D*—H	H···*A*	*D*···*A*	*D*—H···*A*
N1—H1···O1^ii^	0.92 (2)	1.94 (2)	2.815 (3)	159 (2)
O1—H11···N3^iii^	0.99 (3)	1.91 (3)	2.890 (3)	169 (3)
O1—H12···N7^iv^	0.94 (3)	2.07 (3)	2.943 (2)	155 (3)

Symmetry codes: (ii) −*x*+3/2, −*y*+1/2, *z*−1/2; (iii) *x*, −*y*+1, *z*+1/2; (iv) −*x*+3/2, −*y*+1/2, *z*+1/2.
